# TRPM2 Cation Channels Modulate T Cell Effector Functions and Contribute to Autoimmune CNS Inflammation

**DOI:** 10.1371/journal.pone.0047617

**Published:** 2012-10-12

**Authors:** Nico Melzer, Gordon Hicking, Kerstin Göbel, Heinz Wiendl

**Affiliations:** Department of Neurology–Inflammatory Disorders of the Nervous System and Neurooncology, University of Münster, Münster, Germany; Hannover Medical School, Germany

## Abstract

TRPM2, a highly Ca^2+^-permeable member of the transient receptor potential melastatin-related (TRPM) family of cation channels, is expressed in cells of the immune system. We demonstrate firstly that TRPM2 cation channels on T cells critically influence T cell proliferation and proinflammatory cytokine secretion following polyclonal T cell receptor stimulation. Consistently, trpm2-deficient mice exhibited an attenuated clincal phenotype of experimental autoimmune encephalomyelitis (EAE) with reduced inflammatory and demyelinating spinal cord lesions. Importantly, trmp2-deficient T cells were as susceptible as wildtype T cells to oxidative stress-induced cell death as it occurs in inflammatory CNS lesions. This supports the notion that the attenuated EAE phenotype is mainly due to reduced T cell effector functions but unaffected by potential modulation of T cell survival at the site of inflammation. Our findings suggest TRPM2 cation channels as a potential target for treating autoimmune CNS inflammation.

## Introduction

Activation, migration and exertion of effector mechanisms of immune cells critically depend on receptor-operated Ca^2+^-entry from the extracellular space. This takes place via cross-linking of different cell-surface receptors, followed by Ca^2+^ release from intracellular stores via different Ca^2+^ release channels and subsequent Ca^2+^ entry through store-operated plasma membrane Ca^2+^ channels [1], [2].

However, receptor-operated Ca^2+^-entry in immune cells may also occur through non-store operated Ca^2+^-permeable channels [3]. TRPM2 is a highly Ca^2+^-permeable member of the transient receptor potential melastatin-related (TRPM) family of cation channels [4], that is – among others – expressed in various cell types of the innate (dendritic cells [5], monocytes/macrophages [6], [7], [8], CNS microglia [5]) and adaptive (T cells [6], [7], [8] and B cells [9]) immune system. Moreover, functional expression of TRPM2 channels was also demonstrated in CNS neurons [10], [11], [12], [13], [14].

TRPM2 channels are gated by adenosine diphosphate ribose (ADPR) binding to a c-terminal Nudix-like region with pyrophosphatase activity that cleaves ADPR to ribose 5-phosphate and adenosine monophosphate (AMP) [15]. Under physiologic conditions, ADPR predominantly originates form hydrolysis of nicotinamide adenine dinucleotide (NAD^+^) by various plasma membrane-associated and cytosolic glycohydrolases as well as a mitochondrial NADase [16], [17]. Moreover, two other NAD^+^-derived molecules, nicotinic acid adenine dinucleotide phosphate (NAADP) and cyclic ADPR (cADPR), together with Ca^2+^ function as co-agonists facilitating the activation of TRPM2 channels by ADPR [10], [11], [16].

In T cells, cross-linking of cell surface receptors induces a rise of ADPR endogenously generated from NAD^+^ followed by a TRPM2-mediated transmembrane inward current and rise of the intracellular Ca^2+^ concentration [7]. Moreover, cADPR and NAADP were shown to be capable of eliciting TRPM2-mediated transmembrane currents in T cells either by directly binding to the channel or after metabolic conversion to ADPR [6]. Pharmacological inhibition of NAADP signalling in T cells [18] reduced antigen-induced proliferation and cytokine production and ameliorated clinical symptoms of experimental autoimmune encephalomyelitis (EAE, [18]).

In dendritic cells, TRPM2 functions as intracellular Ca^2+^ release channel and is critically involved in differentiation and migration [19].

In CNS neurons, intracellular Ca^2+^ overload and depolarization of the membrane potenital following activation of TRPM2 channels have been implicated in neuronal cell death during oxidative and nitrosative stress [11], [14].

**Figure 1:**
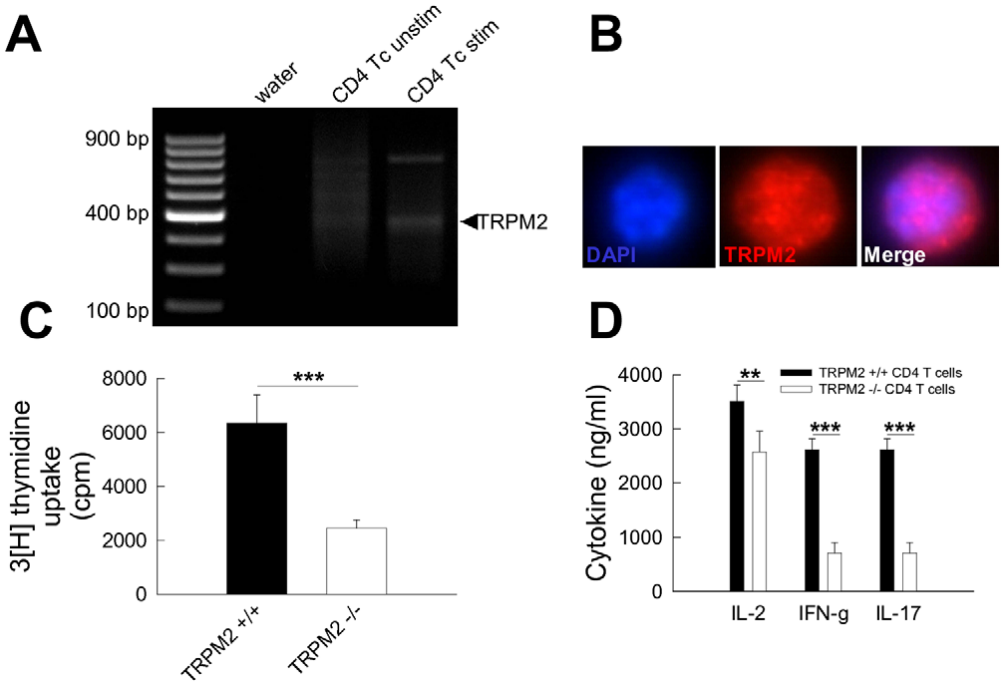
TRPM2 cation channels are upregulated in CD4^+^ T cells upon polyclonal T cell receptor stimulation and critically determine proliferation and proinflammatory cytokine secretion. (**A**) RT-PCR performed with naïve (middle) and anti-CD3/CD28 bead-stimulated (right) wildtype CD4^+^ T cells using murine TRPM2-specific primers revealed upregulation of TRPM2 expression upon stimulation. As negative control water (left) instead of cDNA was used. An experiment representative of 3 independent trials is shown. (**B**) Immunocytochemitry using an antibody specific for the c-terminal portion of the TRPM2 protein (red) revealed positive staining in activated bead-stimulated CD4^+^ T cells. Cell nuclei were counterstained with DAPI (blue). (**C**, **D**) Proliferation as determined by ^3^[H]-thymidine uptake (**C**) and secretion of proinflammatory IL-2, IFN-γ and IL-17 determined by ELISA from the supernatants (**D**) were significantly reduced in CD4^+^ T cells from trpm2-deficient (TRPM2 −/−) as compared to wildtype (TRPM2 +/+) mice following anti-CD3/CD28 bead stimulation at a bead:cell ratio of 1∶4. Moreover, the TRPM2 channel blocker ACA dose-dependently reduced proliferation (filled circles) of bead-stimulated splenocytes (**E**, bead:cell ratio 1∶4) without overt toxic effects on cell viability as assed using flow cytometry for annexin V and propidium iodide (open circles). ACA at a concentration of 100 µM significantly reduced IFN-γ secretion of bead-stimulated splenocytes (**F**, bead:cell ratio 1∶4; n = 3 for all experiments) P-values ≤0.05 were considered significant (*). P-values ≤0.01 and ≤0.001 were considered highly significant (** and ***, respectively).

Here, we study the role of TRPM2 channels in T cell effector functions and autoimmune CNS inflammation using a combination of in vitro approaches and EAE experiments in wildtype and trpm2-deficient mice.

## Materials and Methods

### Mice

Wildtype and trpm2-deficient mice both on a mixed 129/SvJ × C57BL/6J genetic background [20] were kindly provided by Dr. Y. Mori, Kyoto University, Japan. Mice were kept under pathogen-free conditions and had access to food and water ad libidum. All experiments were conducted according to the German law of animal protection and were approved by local authorities (TVA-Nr. 87–51.04.2010. A325, University of Münster, Germany).

**Figure 2:**
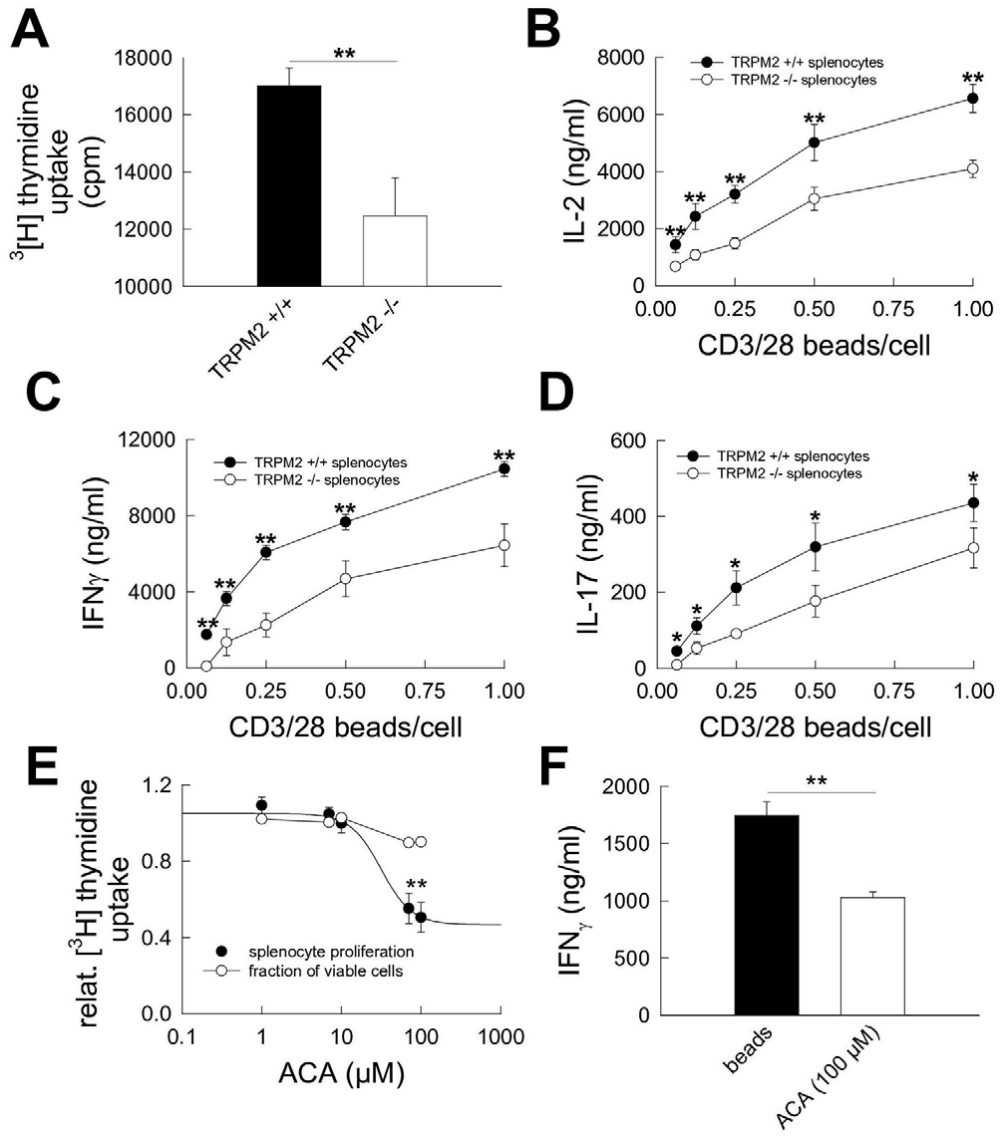
TRPM2 cation channels critically determine proliferation and proinflammatory cytokine secretion following various strengths of polyclonal T cell receptor triggering. (**A–D**) Proliferation as determined by ^3^[H]-thymidine uptake (**A**; bead:cell ratio of 1∶4) and secretion of proinflammatory IL-2 (**B**), IFN-γ (**C**) and IL-17 (**D**) determined by ELISA from the supernatants were significantly reduced in spleenocytes from trpm2-deficient as compared to wildtype mice following anti-CD3/CD28 bead-stimulation for 3 days at various bead:cell ratios (1∶1 to 1∶16; n = 3 for all experiments). P-values ≤0.05 were considered significant (*). P-values ≤0.01 and ≤0.001 were considered highly significant (** and ***, respectively).

### Isolation and culture of splenocytes and T cells

Isolation and culture of splenocytes was performed as described [21]. CD4^+^ T cells were purified from isolated splenocytes using the respective MACS T cell isolation kit (Miltenyi, Bergisch Gladbach, Germany) according to manufacturer's instructions and cultured as described [21].

**Figure 3:**
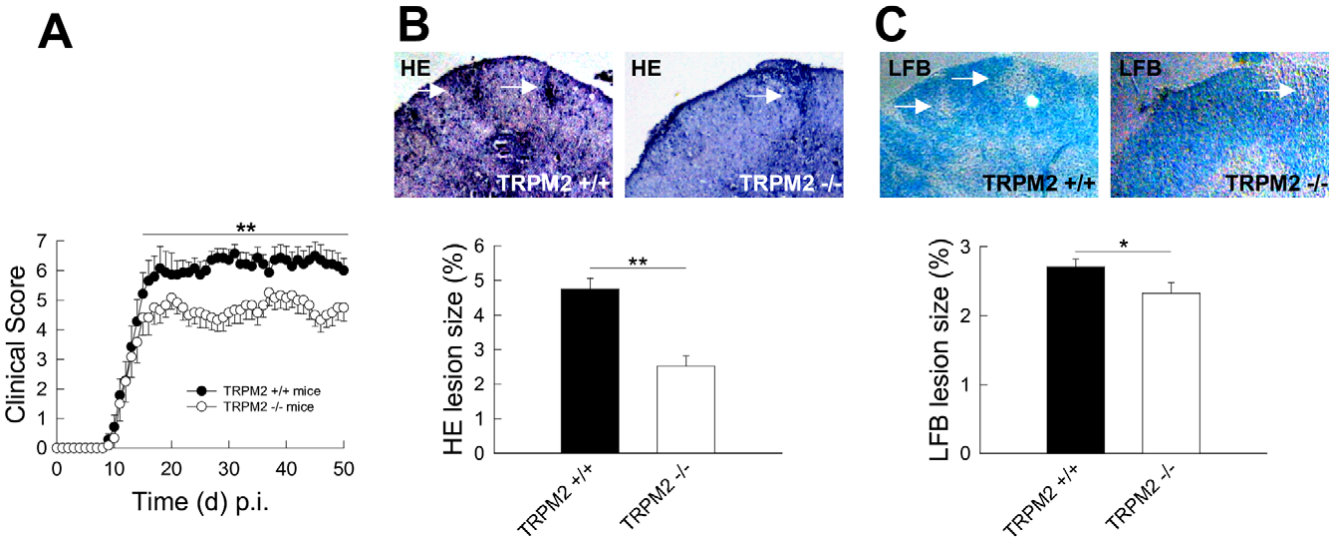
Trpm2-deficient mice exhibit an ameliorated EAE phenotype with reduced inflammatory CNS infiltrates and demyelination. (**A**) Clinical MOG_35–55_ peptide-induced EAE disease score in wildtype and trpm2-deficient mice was examined for 50 days post-immunization (p.i.) and revealed a significantly reduced peak- and residual score in trpm2-deficent as compared to wildtype mice (n = 3 independent trials with 10 mice each for both experimental groups). (**B**, **C**) Representative H&E (**B**) and LFB (**C**) stainig (upper panels) of spinal cord sections from wildtype (left) and trpm2-deficient (right) mice obtained at day 20 post-immunization and quantification of respective leasion sizes (lower panels; n = 2 mice from each of the 3 independent trials representing the mean clinical score of the experimental group at day 20 post-immunization, white arrows indicate lesions). P-values ≤0.05 were considered significant (*). P-values ≤0.01 and ≤0.001 were considered highly significant (** and ***, respectively).

### T cell stimulation, proliferation and cytokine secretion

Splenocytes and purified CD4^+^ T cells from non-immunized mice were stimulated with anti-CD3/CD28 beads (Dynal Biotech, Oslo, Norway) for 72 h at various bead-to-cell ratios. For T cell proliferation analysis, [methyl-3H]-thymidine (1.0 µCi/well; Amersham Biosciences, Buckinghamshire, UK) was added 24 h before the end of incubation. Cells were harvested and [methyl-3H]-thymidine incorporation was measured using a liquid scintillation counter (Packard BioScience, Meriden, USA). For analysis of cytokine secretion, supernatants were analyzed for IL2, IFNγ, and IL17 by ELISA according to the manufacturer's protocol. All experiments were performed as triplicates.

**Figure 4:**
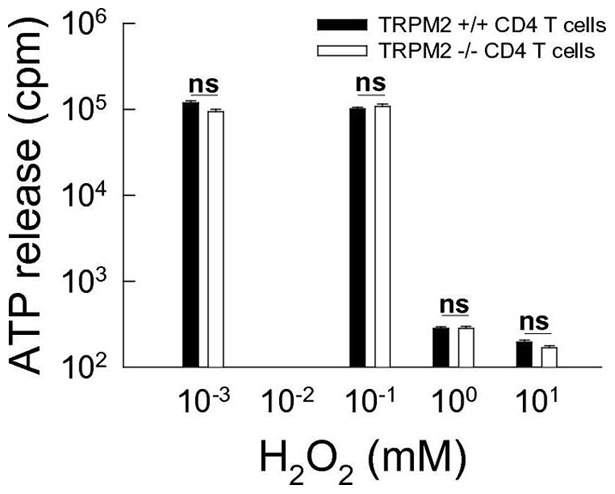
TRPM2 channels do not impact survival of activated CD4^+^ T cells under altered redix conditions. Suvival of activated bead-stimulated CD4^+^ T cells (bead:cell ratio of 1∶4) from wildtype and trpm2-deficient mice under various concentrations of H_2_O_2_ (0.001–10 mM) using the amount of ATP relased following cell lysis as a parameter of cell viability (n = 3; ns  =  not significant).

In a subset of experiments, the TRPM2 channel blocker N-(p-amylcinnamoyl)anthranilic acid (ACA; Sigma) [22], [23] was dissolved in dimethylsulfoxide (DMSO) giving stock solutions of 50 mM. In final solutions, the concentrations of DMSO did not exceed 0.2%, and final DMSO concentrations without ACA served as controls (data not shown).

### RNA preparation and RT-PCR

RNA was purified from naïve and bead-stimulated wildtype CD4^+^ T cells by extraction with peqGold TriFast reagent according to the manufacturer's instructions (peqLab, Erlangen, Germany). cDNA was synthesized from 1 μg of the total RNA using the TaqMan Reverse Transcription Reagents (Applied Biosystems, Darmstadt, Germany). PCR amplification was performed using TRPM2-specific primers under the following conditions: 1 cycle at 94°C for 2 min; 35 or 40 cycles at 94°C for 10 s, 55°C for 10 s, and 72°C for 30 s; and 1 cycle at 72°C for 2 min as described [24]. As negative control water instead of cDNA was used.

### Immunocytochemistry

Immunocytochemical staining was performed on naïve and bead-stimulated wildtype CD4^+^ T cells as described [25]. Cells were placed on coverslips coated with poly-l-lysine (Sigma, Germany) and fixed with 4% paraformaldehyde. Subsequently, cells were blocked with PBS containing 10% horse serum (PAA Laboratories, Cölbe, Germany), 2% bovine serum albumin, and 0.3% Triton X-100 overnight. Next, the primary antibody (rabbit anti-mouse TRPM2, 1∶200, Novus Biologicals, USA) were added and incubated for 1 h. Cells were washed with PBS containing 0.3% Triton X-100 and incubated with secondary antibodies (Cy3-conjugated rabbit anti-goat, 1∶100, Dianova, USA) for another 1 h. Counterstaining of cell nuclei was performed using DAPI (0.5 μg/ml, Merck). Pictures were collected by immunofluorescence microscopy (Axiophot, Zeiss, Jena, Germany). Negative controls without the primary antibody revealed no positive signals (data not shown).

### T cell death analysis

For analysis of H_2_O_2_ induced cell death, 50 000 bead-stimulated CD4^+^ T cells/well were cultured on white 96-well microassay plates (Greiner Bio-One, Frickenhausen, Germany) and incubated for 6 h with different concentrations of H_2_O_2_. Afterwards, the amount of ATP in the supernatant following cell lysis was assessed as a parameter of cell viability using the ATPLite™ Luminescence Assay System (PerkinElmer, Rodgau-Jügesheim, Germany) according to the manufacturer's instructions. Luminescence was measured on a Topcount NXT (PerkinElmer, Rodgau, Germany). All experiments were performed as triplicates.

In a subset of experiments, cell death analysis was performed using flow cytometry for annexin V and propidium iodide. The viable cell fraction was considered to represent the fraction of annexin V/propidium iodide double-negative cells as described [21].

### Experimental autoimmune encephalomyelitis (EAE)

Induction and clinical evaluation of myelin oligodendrocyte glycoprotein (MOG_35–55_) peptide-induced EAE was performed as described [21].

### Histopathology

On day 20 of the EAE experiments, mice were sacrificed and transcardially perfused with phosphate-buffered saline (PBS). Spinal cords were carefully excised from the brainstem to lumbar region, fixed in 4% paraformaldehyde (PFA) in PBS for 2 h and then embedded in Tissue-Tek OCT compound (Miles Laboratories, Elkhart, USA). Ensuring that the same regions were analysed for all mice, 10-μm cross sections of the beginning of the cervical enlargement were cut [26]. H&E and Luxol fast blue (LFB) stainings were performed on paraformaldehyde-fixed spinal cord cryosections according to standard procedures [2]. Spinal cord inflammatory infiltrates and demyelination were assessed by quantifying the respective lesion area relative to the total section area in 100 H&E and LFB stained sections randomly chosen from the cervical and lumbal part of the spinal cord, respectively.

### Statistical analysis

All results are presented as mean ± sem. Statistical analysis was performed using the student's t-test for normally distributed datasets or a Wilcoxon rank sum test for non-normally distributed datasets. A one-way ANOVA was used in case of multiple comparisons using SigmaPlot 11.0 software (Sigma Plot). P-values ≤0.05 were considered significant (*). P-values ≤0.01 and ≤0.001 were considered highly significant (** and ***, respectively).

## Results

### TRPM2 channel expression is upregulated in CD4^+^ T cells following polyclonal T cell receptor stimulation *in vitro*

To assess the expression and regulation of TRPM2 cation channels, isolated naïve murine CD4^+^ T cells were polyclonally stimulated for 3 days *in vitro* using anti-CD3/CD28-coated beads at a bead:cell ratio of 1∶4. RT-PCR revealed an upregulation of TRPM2 message following bead-stimulation after 3 days (**Fig. 1** **A**). Moreover, immunocytochemistry using an antibody specific for the c-terminal portion of the TRPM2 protein revealed positive staining in activated bead-stimulated CD4^+^ T cells (**Fig. 1** **B**).

### CD4^+^ T cells from TRPM2-deficient mice show reduced proliferation and proinflammatory cytokine secretion upon polyclonal T cell receptor stimulation *in vitro*

To assess the impact of TRPM2 channels on T cell effector functions, we first determined proliferation and and secretion of various proinflammatory cytokines in CD4^+^ T cells isolated from wildtype and trpm2-deficient mice following bead-stimulation for 3 days at a fixed bead:cell ratio of 1∶4. CD4^+^ T cells from trpm2-deficient mice showed significantly reduced proliferation (**Fig. 1** **C**) and secretion of different effector cytokines such as IL-2, IFN-γ, and IL-17 (**Fig. 1** **D**) as compared to those from wildtype mice.

To assess this effect in the whole population of CD4^+^ and CD8^+^ T cells, we measured proliferation and and cytokine secretion in splenocytes from wildtype and trpm2-deficient mice following bead-stimulation for 3 days. To mimick different strengths of T cell receptor triggering, the bead:cell ratio was varied between of 1∶1 and 1∶16 during these experiments. Again, splenoytes from trpm2-deficient mice displayed significantly reduced proliferation (**Fig. 2** **A**, bead:cell ratio 1∶4) and secretion of IL-2 (**Fig. 2** **B**), IFN-γ (**Fig. 2** **C**), and IL-17 (**Fig. 2** **D**) as compared to those from wildtype mice under all experimental conditions.

The TRPM2 channel blocker N-(p-amylcinnamoyl)anthranilic acid (ACA) is known to inhibit TRPM2 channel activity at concentrations between 10 and 100 µM [22], [23]. Hence, we aimed to reproduce the effect of TRPM2-deficiency on T cell effector functions using different concentrations of ACA. Indeed, ACA dose-dependently reduced proliferation of bead-stimulated splenocytes (**Fig. 1** **. E**, bead:cell ratio 1∶4) without overt toxic effects on cell viability as assed using flow cytometry for annexin V and propidium iodide. Consistently, ACA at a concentration of 100 µM significantly reduced IFN-γ secretion of bead-stimulated splenocytes (**Fig. 1** **. F**, bead:cell ratio 1∶4). Thus, genetic deficency and pharmacological inhibition of TRPM2 channels dampened proliferation and proinflammatory cytokine secretion in splenocytes following polyclonal T cell receptor stimulation *in vitro*.

### Trpm2-deficient mice exhibit an ameliorated EAE phenotype with reduced inflammatory CNS infiltrates and demyelination in vivo

Given these effects of TRPM2 channels on T cell effector function, we performed MOG_35–55_-peptide induced EAE experiments comparing wildtype and trpm2-deficient mice (**Fig. 3** **A–C**). As compared to wildtype mice, trpm2-deficient mice displayed an attenuated EAE phenotype: although the mean time until disease onset was identical between wildtype and trpm2-deficient mice (9 days vs. 9 days), trpm2-deficient mice displayed a delayed time to peak disease (20 days vs. 18 days). Moreover, trpm2-deficient mice displayed significantly reduced peak- and residual disease scores (**Fig. 3** **A**).

Consistent with the ameliorated clinical phenotype, H&E (**Fig. 3** **B**) and LFB (**Fig. 3** **C**) stainig of spinal cord sections from EAE mice representative of the mean clinical score of the respective experimental group at day 20 post-immunization displayed reduced inflammation (**Fig. 3** **B**) and demyelination (**Fig. 3** **C**) in the spinal cord of trpm2-deficient as compared to wildtype mice.

### TRPM2 channels do not impact the survival of activated CD4^+^ T cells under altered redox conditions

Given the potential role of TRPM2 channels in cell death of T cells in an inflammatory CNS lesion due to its activation by reactive oxygen species [27] we tested suvival of activated bead-stimulated CD4^+^ T cells from wildtype and trpm2-deficient mice under various concentrations of H_2_O_2_ (0.001–10 mM; **Fig. 4**) using the amount of ATP relased following cell lysis as a parameter of cell viability. Activated CD4^+^ T cell viability did not differ between wildtype and trpm2-deficient mice under all experimental conditions refruiting a role of TRPM2 channels in T cell death under altered redox conditions.

## Discussion

Taken together, we firstly demonstrate here a role for TRPM2 cation channels in T cell proliferation and proinflammatory cytokine secretion following polyclonal T cell receptor stimulation. The effects of TRPM2-deficiency on T cell effector function could be mimicked by application of the TRPM2 channel blocker N-(p-amylcinnamoyl)anthranilic acid (ACA; [22], [23]) providing an opportunity for pharmacological modulation of TRPM2 channel function in T cells. Moreover, TRPM2 channels have been shown to dramatically impact the maturation and chemokine-activated directional migration of dendritic cells, which function as antigen-presenting cells [19]. Both findings, strongly imply an attenuated clincal EAE phenotype with reduced inflammatory and demyelinating spinal cord lesions in trpm2-deficient mice as observed.

Moreover, TRPM2 expression within the CNS is in large parts attributed to microglia, the CNS resident monocyte-lineage derived cell type [5]. In microglia cells TRPM2 currents and Ca^2+^-entry could be evoked by triggering of toll-like receptors (LPS) and cytokine receptors (TNFα) as well as by intracellular ADPR and play a pivotal role in the activation of these cells [5]. Hence, it is very likely that reduced activation of CNS microglia contributes to the attenuated clinical EAE phenotype in trpm2-deficient mice.

There are also several lines of evidence that TRPM2 channels are expressed in CNS neurons: TRPM2-mediated currents evoked either by extracellular application of H_2_O_2_ or OONO^-^ or by intracellular perfusion with NAD^+^-derived second messengers like ADPR have been identified in cultured striatal neurons [10], [14], acutely isolated cortical neurons [11] and hippocampal neurons in culture and slice preparations [12], [13]. These currents and the associated Ca^2+^ overload have been implicated in neuronal cell death during oxidative and nitrosative stress [11], [14], which is a dominant mechanism of neuronal cell death in EAE. Hence, lack of TRPM2 channels is very likely to protect neurons and axons from degeneration during EAE and thus may also contribute to the ameliorated clinical EAE phenotype. These findings and considerations strongly suggest that pharmacological inhibition of TRPM2 cation channels might be regarded as a strategy for treating autoimmune CNS inflammation.

However, TRPM2 channels have been suggested to confer susceptibility to cell death following changes in redox status as they occur in inflammatory CNS lesions [27]. However, under conditions of oxidative stress, we did not observe sustained survival of trpm2-deficient T cells as compared to wildtype T cells. This is consistent with the unaltered number of T cells in wildtype and trpm2-deficient mice in a model of colon inflammation [20] and suggests that TRPM2 channels do not overtly affect lymphocyte survival at the site of inflammation.

Hence, inhibition of TRPM2 channels in autoimmune inflammatory CNS disorders likely dampens the adaptive T cell-mediated immune response without favouring prolonged T cell survival and inflammatory tissue damage. Morevoer, TRPM2 channel inhibtion likely attenuates microglia activation and neurodegenration during autoimmune CNS inflammation.

## References

[1] Feske S (2007) Calcium signalling in lymphocyte activation and disease. Nat Rev Immunol 7: 690–702. 17703229 10.1038/nri2152

[2] Schuhmann MK, Stegner D, Berna-Erro A, Bittner S, Braun A, et al (2010) Stromal interaction molecules 1 and 2 are key regulators of autoreactive T cell activation in murine autoimmune central nervous system inflammation. J Immunol 184: 1536–1542. 20028655 10.4049/jimmunol.0902161

[3] Vig M, Kinet JP (2009) Calcium signaling in immune cells. Nat Immunol 10: 21–27. 19088738 10.1038/ni.f.220 PMC2877033

[4] Harteneck C (2005) Function and pharmacology of TRPM cation channels. Naunyn Schmiedebergs Arch Pharmacol 371: 307–314. 15843919 10.1007/s00210-005-1034-x

[5] Kraft R, Grimm C, Grosse K, Hoffmann A, Sauerbruch S, et al (2004) Hydrogen peroxide and ADP-ribose induce TRPM2-mediated calcium influx and cation currents in microglia. Am J Physiol Cell Physiol 286: C129–137. 14512294 10.1152/ajpcell.00331.2003

[6] Beck A, Kolisek M, Bagley LA, Fleig A, Penner R (2006) Nicotinic acid adenine dinucleotide phosphate and cyclic ADP-ribose regulate TRPM2 channels in T lymphocytes. Faseb J 20: 962–964. 16585058 10.1096/fj.05-5538fje

[7] Gasser A, Glassmeier G, Fliegert R, Langhorst MF, Meinke S, et al (2006) Activation of T cell calcium influx by the second messenger ADP-ribose. J Biol Chem 281: 2489–2496. 16316998 10.1074/jbc.M506525200

[8] Sano Y, Inamura K, Miyake A, Mochizuki S, Yokoi H, et al (2001) Immunocyte Ca2+ influx system mediated by LTRPC2. Science 293: 1327–1330. 11509734 10.1126/science.1062473

[9] Buelow B, Song Y, Scharenberg AM (2008) The Poly(ADP-ribose) polymerase PARP-1 is required for oxidative stress-induced TRPM2 activation in lymphocytes. J Biol Chem 283: 24571–24583. 18599483 10.1074/jbc.M802673200 PMC3259813

[10] Hill K, Tigue NJ, Kelsell RE, Benham CD, McNulty S, et al (2006) Characterisation of recombinant rat TRPM2 and a TRPM2-like conductance in cultured rat striatal neurones. Neuropharmacology 50: 89–97. 16260005 10.1016/j.neuropharm.2005.08.021

[11] Kaneko S, Kawakami S, Hara Y, Wakamori M, Itoh E, et al (2006) A critical role of TRPM2 in neuronal cell death by hydrogen peroxide. J Pharmacol Sci 101: 66–76. 16651700 10.1254/jphs.fp0060128

[12] Lipski J, Park TI, Li D, Lee SC, Trevarton AJ, et al (2006) Involvement of TRP-like channels in the acute ischemic response of hippocampal CA1 neurons in brain slices. Brain Res 1077: 187–199. 16483552 10.1016/j.brainres.2006.01.016

[13] Olah ME, Jackson MF, Li H, Perez Y, Sun HS, et al (2009) Ca2+-dependent induction of TRPM2 currents in hippocampal neurons. J Physiol 587: 965–979. 19124544 10.1113/jphysiol.2008.162289 PMC2673769

[14] Smith MA, Herson PS, Lee K, Pinnock RD, Ashford ML (2003) Hydrogen-peroxide-induced toxicity of rat striatal neurones involves activation of a non-selective cation channel. J Physiol 547: 417–425. 12562896 10.1113/jphysiol.2002.034561 PMC2342643

[15] Perraud AL, Fleig A, Dunn CA, Bagley LA, Launay P, et al (2001) ADP-ribose gating of the calcium-permeable LTRPC2 channel revealed by Nudix motif homology. Nature 411: 595–599. 11385575 10.1038/35079100

[16] Lund FE (2006) Signaling properties of CD38 in the mouse immune system: enzyme-dependent and -independent roles in immunity. Mol Med 12: 328–333. 17380200 10.2119/2006-00099.Lund PMC1829203

[17] Malavasi F, Deaglio S, Ferrero E, Funaro A, Sancho J, et al (2006) CD38 and CD157 as receptors of the immune system: a bridge between innate and adaptive immunity. Mol Med 12: 334–341. 17380201 10.2119/2006-00094.Malavasi PMC1829205

[18] Dammermann W, Zhang B, Nebel M, Cordiglieri C, Odoardi F, et al (2009) NAADP-mediated Ca2+ signaling via type 1 ryanodine receptor in T cells revealed by a synthetic NAADP antagonist. Proc Natl Acad Sci U S A 106: 10678–10683. 19541638 10.1073/pnas.0809997106 PMC2697110

[19] Sumoza-Toledo A, Lange I, Cortado H, Bhagat H, Mori Y, et al (2011) Dendritic cell maturation and chemotaxis is regulated by TRPM2-mediated lysosomal Ca2+ release. FASEB J 25: 3529–3542. 21753080 10.1096/fj.10-178483 PMC3177582

[20] Yamamoto S, Shimizu S, Kiyonaka S, Takahashi N, Wajima T, et al (2008) TRPM2-mediated Ca2+influx induces chemokine production in monocytes that aggravates inflammatory neutrophil infiltration. Nat Med 14: 738–747. 18542050 10.1038/nm1758 PMC2789807

[21] Melzer N, Meuth SG, Torres-Salazar D, Bittner S, Zozulya AL, et al (2008) A beta-lactam antibiotic dampens excitotoxic inflammatory CNS damage in a mouse model of multiple sclerosis. PLoS ONE 3: e3149. 18773080 10.1371/journal.pone.0003149 PMC2522272

[22] Bari MR, Akbar S, Eweida M, Kuhn FJ, Gustafsson AJ, et al (2009) H2O2-induced Ca2+ influx and its inhibition by N-(p-amylcinnamoyl) anthranilic acid in the beta-cells: involvement of TRPM2 channels. J Cell Mol Med 13: 3260–3267. 19382906 10.1111/j.1582-4934.2009.00737.x PMC4516483

[23] Kraft R, Grimm C, Frenzel H, Harteneck C (2006) Inhibition of TRPM2 cation channels by N-(p-amylcinnamoyl)anthranilic acid. Br J Pharmacol 148: 264–273. 16604090 10.1038/sj.bjp.0706739 PMC1751561

[24] Inada H, Iida T, Tominaga M (2006) Different expression patterns of TRP genes in murine B and T lymphocytes. Biochem Biophys Res Commun 350: 762–767. 17027915 10.1016/j.bbrc.2006.09.111

[25] Meuth SG, Bittner S, Meuth P, Simon OJ, Budde T, et al (2008) TWIK-related acid-sensitive K+ channel 1 (TASK1) and TASK3 critically influence T lymphocyte effector functions. J Biol Chem 283: 14559–14570. 18375952 10.1074/jbc.M800637200

[26] Bittner S, Meuth SG, Gobel K, Melzer N, Herrmann AM, et al (2009) TASK1 modulates inflammation and neurodegeneration in autoimmune inflammation of the central nervous system. Brain 132: 2501–2516. 19570851 10.1093/brain/awp163 PMC3031313

[27] Hara Y, Wakamori M, Ishii M, Maeno E, Nishida M, et al (2002) LTRPC2 Ca2+-permeable channel activated by changes in redox status confers susceptibility to cell death. Mol Cell 9: 163–173. 11804595 10.1016/s1097-2765(01)00438-5

